# Supplementary material for: Efficient protein production by yeast requires global tuning of metabolism
Source: Nat Commun. 2017 Oct 25;8:1131. doi: 10.1038/s41467-017-00999-2 (PMC5656615; doi:10.1038/s41467-017-00999-2)
Supplement: Supplementary file 1 — Supplementary Information [file 41467_2017_999_MOESM1_ESM.docx]

Intracellular α-amylase (%)

α-amylase yield (U g-DCW^-1^)


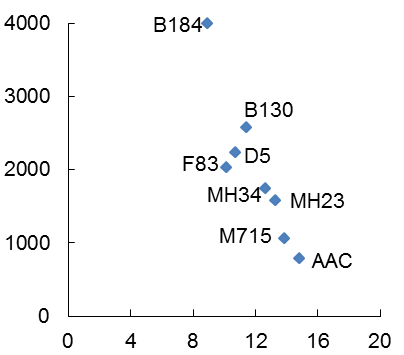


Supplementary Figure 1. (a-c) Physiological parameters of the mutant strains are plotted in graphs. (d) Intracellular percentage of α-amylase at the end of batch cultures. (e) Cell growth in batch cultures. Data shown are mean values ± standard deviations of triplicates or quadruplicates.

**e**

DCW (g L^-1^)

Time (h)


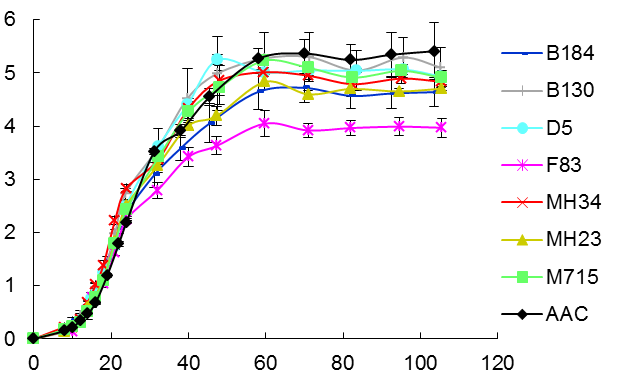


Specific α-amylase production rate (U g-DCW^-1^ h^-1^)


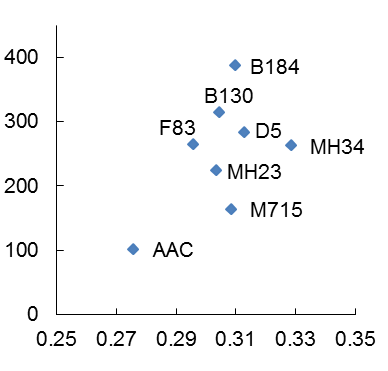


Maximum specific growth rate (h^-1^)

Specific glucose uptake rate

(g g-DCW^-1^ h^-1^)

Specific ethanol production rate

(g g-DCW^-1^ h^-1^)


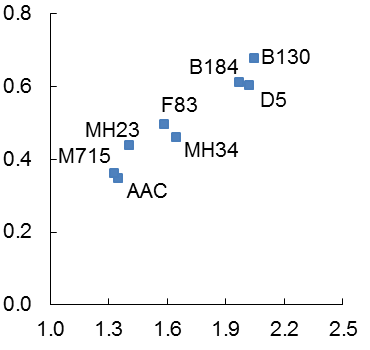


Specific glycerol production rate (g g-DCW^-1^ h^-1^)

Specific acetate production rate

(g g-DCW^-1^ h^-1^)


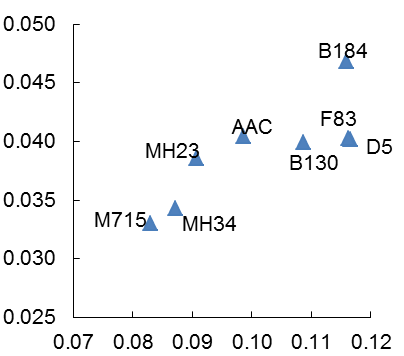


**a**

**b**

**c**

**d**


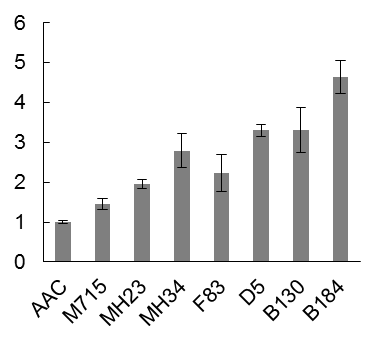


Relative α-amylase yield

Exponential phase

**a**

Supplementary Figure 2. (a) Relative α-amylase yield in mutant strains compared with the reference strain AAC at the 40 h of cultivation. (b) Relative α-amylase yield in mutant strains compared with the reference strain AAC at the end of cultivation. (c) Dissolved oxygen (DO) level at the RNA sampling time points. (d) Percentage of total α-amylase in total cell protein at the end of cultivation. The coefficient of 450 mg cell protein per g-DCW was used for calculation of total cell protein from DCW. Data shown are mean values ± standard deviations of triplicates or quadruplicates.


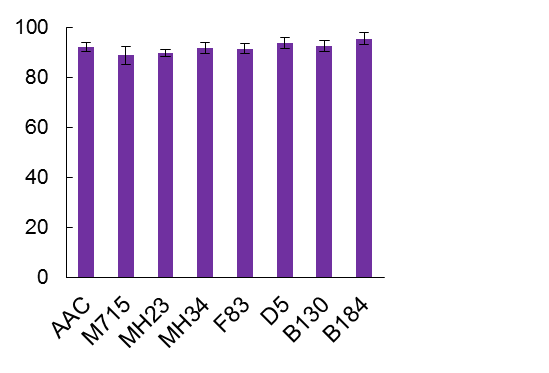


DO level (%)

**c**


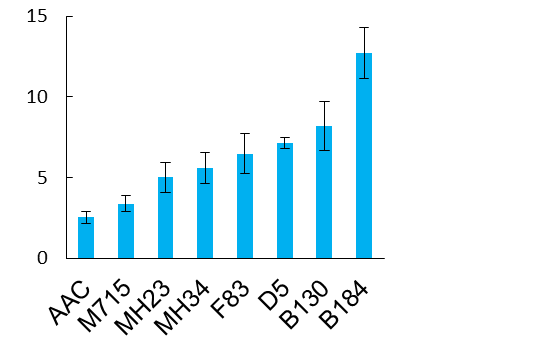


α-amylase/Total cell protein (%)

**d**


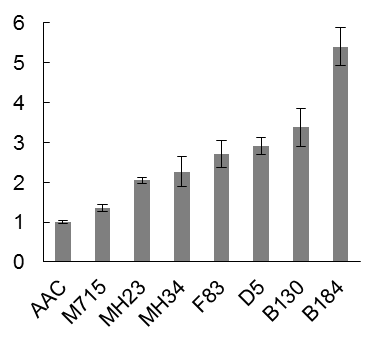


Relative α-amylase yield

Cultivation end

**b**


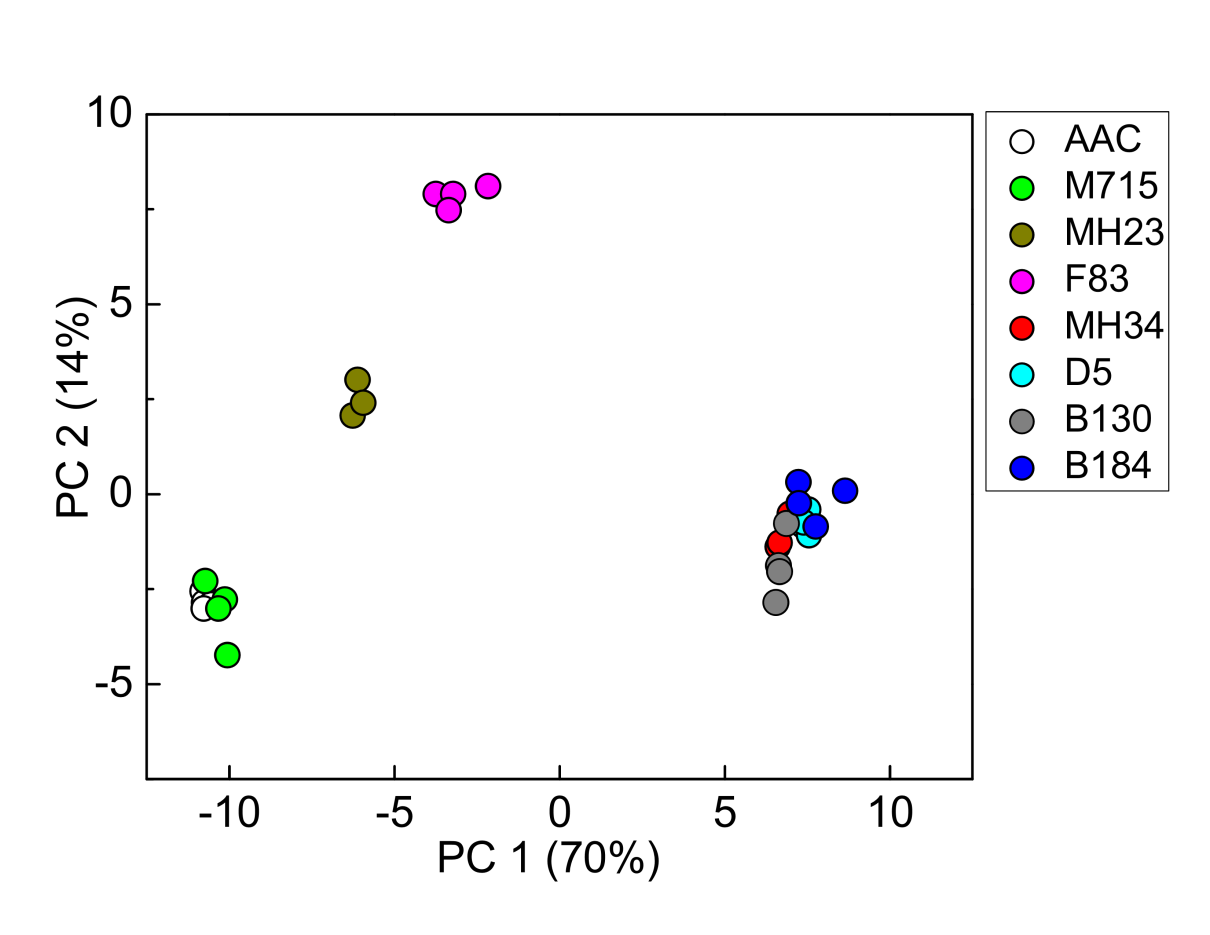


2

1

3

Supplementary Figure 3. Principal component analysis (PCA) was performed by using expression profiles. Strains were classified into three groups: group 1 contained AAC and M715; group 2 contained MH23 and F83; group 3 contained MH34, D5, B130 and B184.

P-adj < 0.05 & abs (log_2_ Fold change) > 1

P-adj ≥ 0.05 & abs (log_2_ Fold change) > 1

P-adj < 0.05 & abs (log_2_ Fold change) ≤ 1

P-adj ≥ 0.05 & abs (log_2_ Fold change) ≤ 1


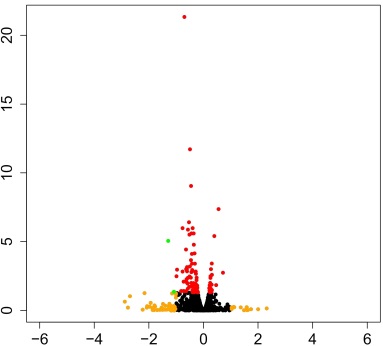


log_2_ Fold change

-log_10_ P-adj

M715 vs. AAC


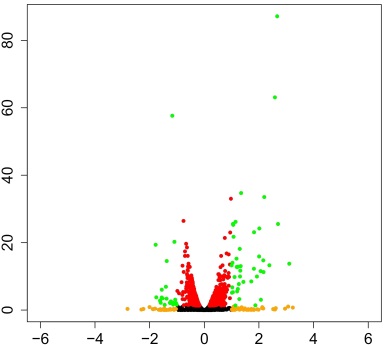


MH23 vs. AAC

log_2_ Fold change

-log_10_ P-adj

F83 vs. AAC


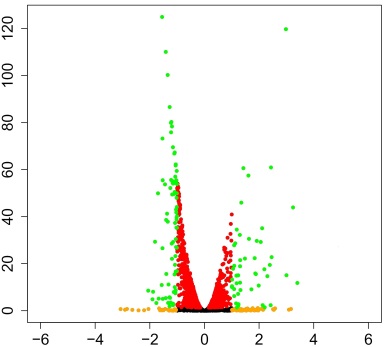


log_2_ Fold change

-log_10_ P-adj

MH34 vs. AAC


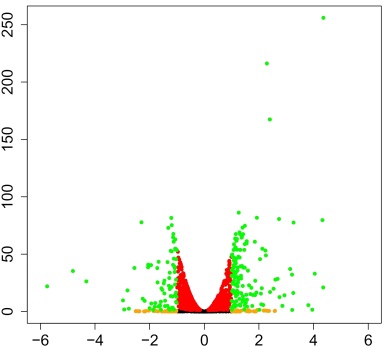


log_2_ Fold change

-log_10_ P-adj

D5 vs. AAC


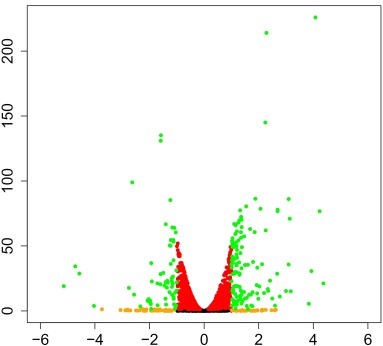


log_2_ Fold change

-log_10_ P-adj


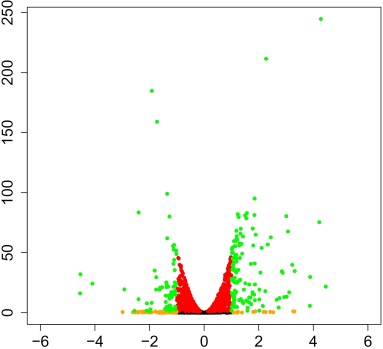


B130 vs. AAC

log_2_ Fold change

-log_10_ P-adj

B184 vs. AAC


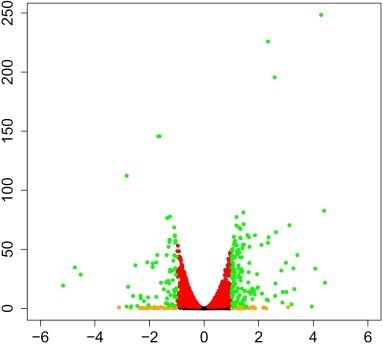


log_2_ Fold change

-log_10_ P-adj

**b**

**a**

Supplementary Figure 4. Different gene expression levels in mutant strains. (a) Hierarchical clustering of different expression genes. (b)Transcriptional changes are presented in volcano plot. Adjusted p values (P-adj) were calculated using Benjamini-Hochberg method.

log_2_ Fold change


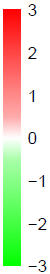


-3

0

3


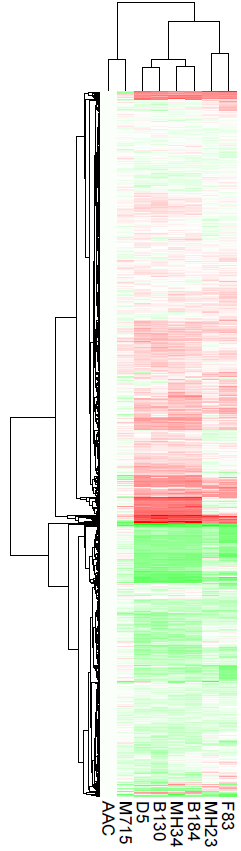


AAC

M715

D5

B130

MH34

B184

MH23

F83

TFs

MH23

F83

MH34

D5

B130

B184

MH23

F83

MH34

D5

B130

B184

Directional log_10_ p-value

16

-16

0

down

up


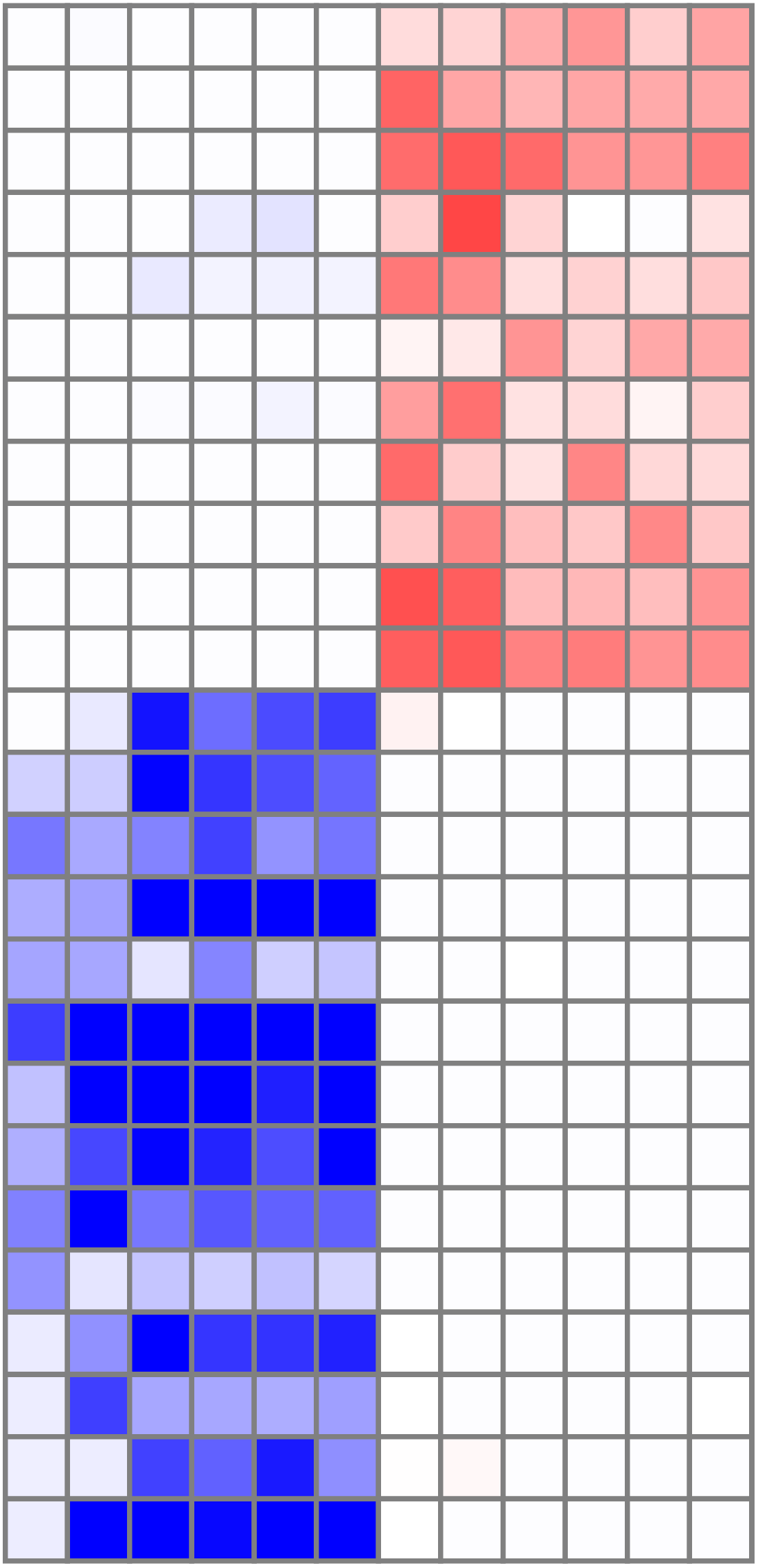


*MBP1*

*MIG2*

*MSS11*

*NNF2*

*ROX1*

*SIR2*

*SWI4*

*TEC1*

*TUP1*

*UPC2*

*YOX1*

*ADR1*

*BAS1*

*GCR1*

*HAP1*

*HAP2*

*HAP3*

*HAP4*

*HAP5*

*HIR3*

*HST1*

*MSN2*

*NRG2*

*OPI1*

*SUT1*

Supplementary Figure 5. Reporter transcription factors (TFs) analysis when genes on the chromosome III in strains MH34, D5, B130 and B184 were removed. The top 5 scored reporter TFs for each strain in distinct-directional up class (red) and distinct-directional down class (blue) are chosen and presented by their significance.


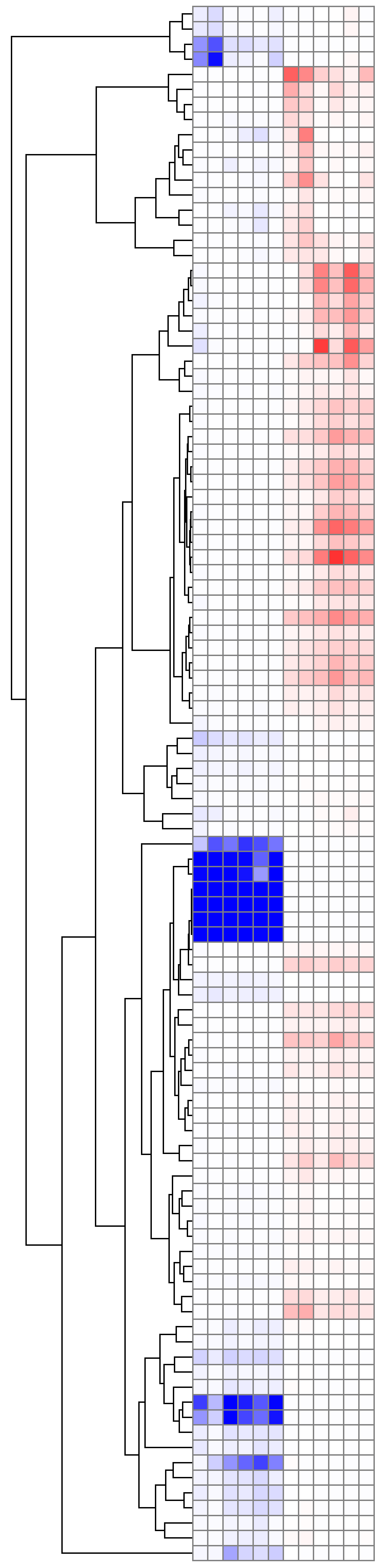


down

up

MH23

F83

MH34

D5

B130

B184

MH23

F83

MH34

D5

B130

B184

translational initiation

regulation of translation

protein complex biogenesis

tRNA aminoacylation for protein translation

biological_process

transcription from RNA polymerase II promoter

signaling

response to osmotic stress

lipid metabolic process

Golgi vesicle transport

carbohydrate metabolic process

protein glycosylation

proteolysis involved in cellular protein catabolic process

response to chemical

cell wall organization or biogenesis

protein lipidation

amino acid transport

rRNA processing

ribosomal small subunit biogenesis

ribosome assembly

organelle assembly

ribosomal large subunit biogenesis

cytoplasmic translation

nuclear transport

ribosomal subunit export from nucleus

nucleobase-containing compound transport

cytoskeleton organization

chromosome segregation

chromatin organization

histone modification

DNA repair

cellular response to DNA damage stimulus

regulation of organelle organization

DNA replication

organelle fission

regulation of DNA metabolic process

mitotic cell cycle

cytokinesis

DNA recombination

telomere organization

regulation of cell cycle

cell budding

nucleus organization

meiotic cell cycle

conjugation

invasive growth in response to glucose limitation

pseudohyphal growth

protein acylation

protein folding

RNA modification

tRNA processing

protein alkylation

organelle inheritance

translational elongation

peptidyl-amino acid modification

transmembrane transport

mitochondrial translation

cellular amino acid metabolic process

mitochondrion organization

cellular respiration

generation of precursor metabolites and energy

nucleobase-containing small molecule metabolic process

snoRNA processing

regulation of protein modification process

protein targeting

protein maturation

DNA-templated transcription, elongation

RNA catabolic process

protein phosphorylation

transposition

response to starvation

cell morphogenesis

DNA-templated transcription, termination

DNA-templated transcription, initiation

regulation of transport

protein modification by small protein conjugation or removal

oligosaccharide metabolic process

endosomal transport

transcription from RNA polymerase III promoter

vesicle organization

protein dephosphorylation

endocytosis

RNA splicing

mRNA processing

not_yet_annotated

transcription from RNA polymerase I promoter

carbohydrate transport

lipid transport

response to heat

response to oxidative stress

exocytosis

other

cofactor metabolic process

monocarboxylic acid metabolic process

membrane invagination

organelle fusion

ion transport

peroxisome organization

membrane fusion

vacuole organization

sporulation

cellular ion homeostasis

vitamin metabolic process

GO terms

Directional log_10_ p-value

16

-16

0

Supplementary Figure 6. Reporter GO terms analysis. The distinct-directional up class (red) and distinct-directional down class (blue) are presented by their significance.


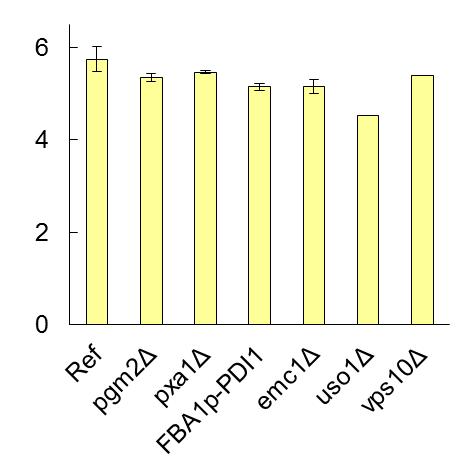


DCW (g L^-1^)

Supplementary Figure 7. The impact of gene on α-amylase secretion. (a) Relative α-amylase yield of engineered strains, AACK was used as the reference strain. (b) Biomass of engineered strains. Gene deletion was carrired out by using amdS marker. *PDI1* was overexpressed by strong promoter FBA1p replacement. Data shown are mean values ± standard deviations of duplicates.

α-amylase yield (Fold)


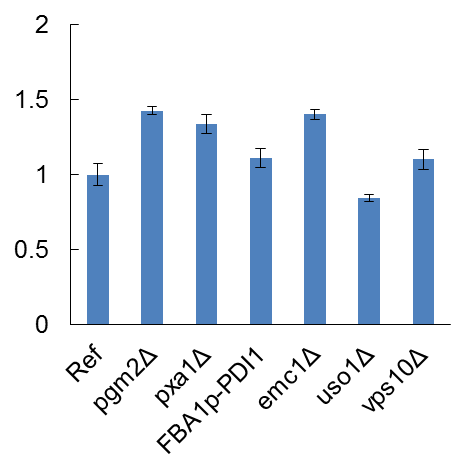


**a**

**b**


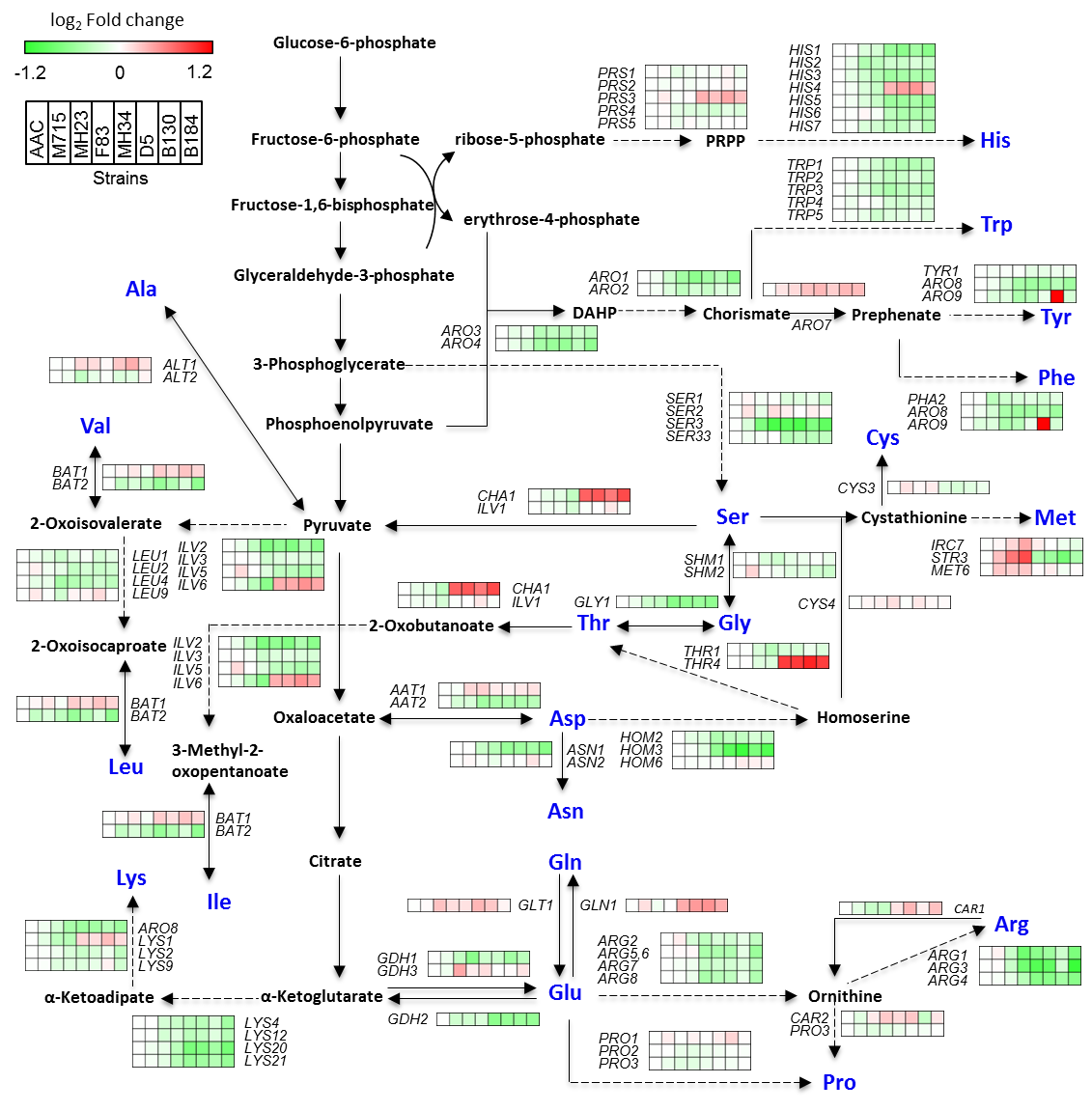


Supplementary Figure 8. Transcriptional changes of genes involved in amino acid biosynthesis.

Supplementary Figure 9. Transcriptional changes of amino acid transporter genes. Genes marked in blue is transcriptionally controlled by the Ssy1p-Ptr3p-Ssy5p (SPS) sensor, which senses external amino acid concentration. Genes marked in green is under nitrogen regulation.

**Cytosol**

**Nucleus**

**Mitochondria**

**ER**

**Vacuole**

*GNP1*

**Golgi**

AAC

M715

MH23

F83

MH34

D5

B130

B184

Strains

log_2_ Fold change

1.2

-1.2

0


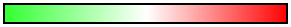


*AGP3*

*ALP1*

*HIP1*

*LYP1*

*MMP1*

*MUP1*

*MUP3*


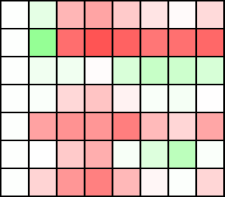


*AGP1*

*BAP2*

*BAP3*

*TAT1*

*TAT2*


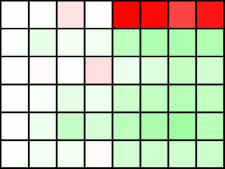


*CAN1*

*DIP5*

*GAP1*

*PUT4*


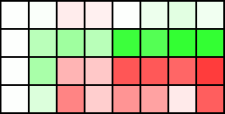


*AGP2*

*SAM3*

*SSY1*

*UGA4*

*HNM1*

*YCT1*


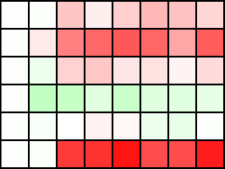


*AGC1*

*CRC1*

*ORT1*


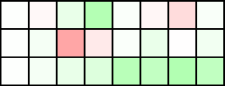


*AVT1*

*AVT3*

*AVT4*

*AVT6*


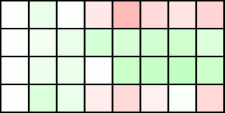


*AQR1*

*VBA1*

*VBA2*

*ATG22*


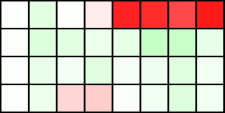


*YPQ1*

*YPQ2*

*ERS1*


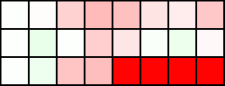


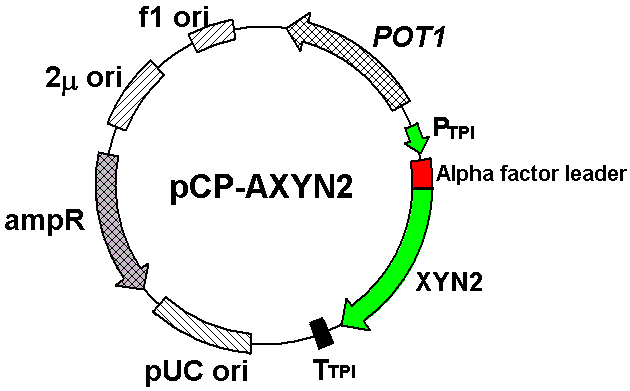

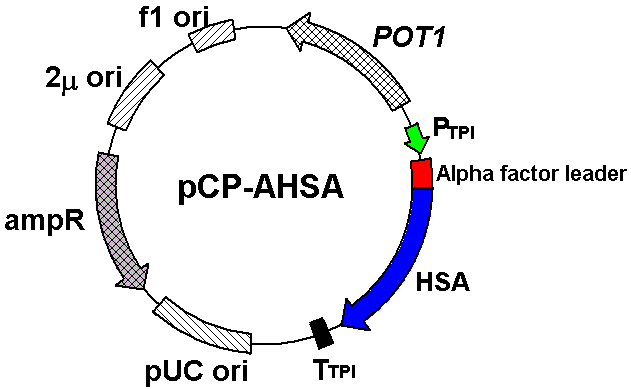


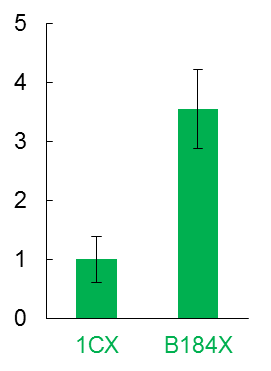


Relative XYN yield (Fold)


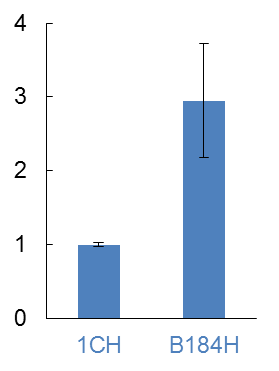


Relative HSA yield (Fold)

Supplementary Figure 10. Secretion of human serum albumin (HSA) and *Trichoderma reesei* endo-1,4-beta-xylanase II (XYN) increased in mutant strain B184 compared with the reference strain CEN.PK 530.1C. (a) and (b) Synthesized human serum albumin (HSA) gene and *T. reesei* endo-1,4-beta-xylanase II gene were cloned in plasmid CPOTud. The alpha factor leader was used as the secretory signal peptide. (c) SDS/PAGE analysis of HSA and XYN in the supernatant from different strains. The same amount (18 μL) of supernatant was loaded on the gel. NC: strain with empty plasmid as negative control. M: protein marker. 1CH: secretion of HSA by reference strain CEN.PK 530.1C. 1CX: secretion of XYN by reference strain CEN.PK 530.1C. B184H: secretion of HSA by mutant strain B184. B184X: secretion of XYN by mutant strain B184. (d and e) Relative secretion yield of HSA and XYN was higher in strain B184 compared with the reference strain. Data shown are mean values ± standard deviations of duplicates.

**c**

**e**

**d**

**b**

**a**


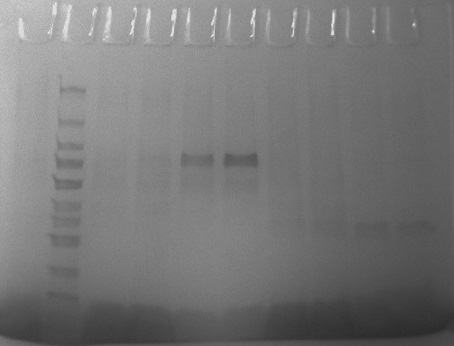


HSA

XYN

70 KDa

25 KDa

NC

M

1CH

1CH.

B184H

B184H

1CX

1CX

B184X

B184X

HSA

XYN


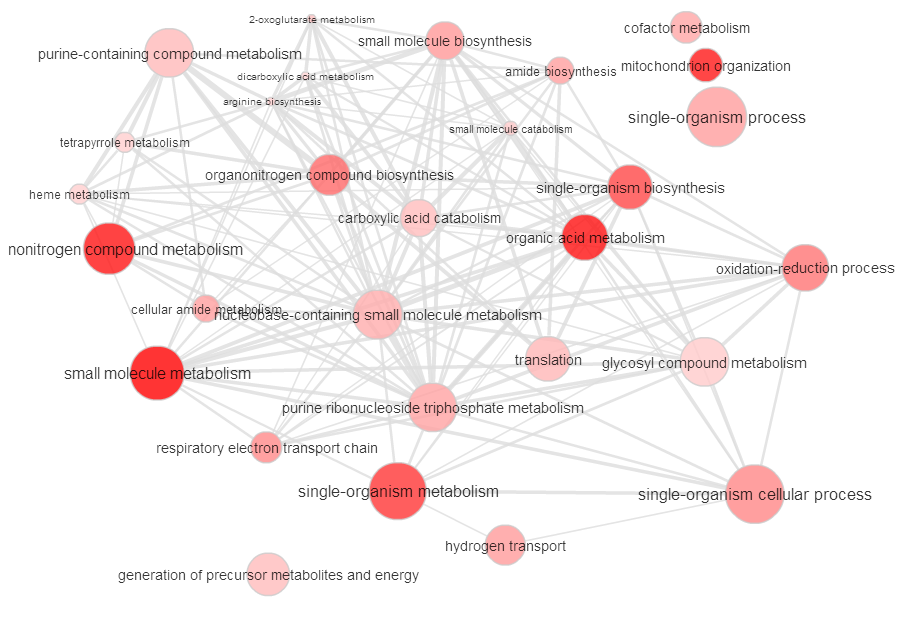


Supplementary Figure 11. GO bioprocess enrichment of most down-regulation genes (P-adj< 0.05 (Benjamini-Hochberg method) and log_2_ Fold change < -0.5) in strain B184.

**c**

log_2_ Fold change

1.2

-1.2

0


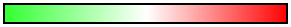


AAC

M715

MH23

F83

MH34

D5

B130

B184


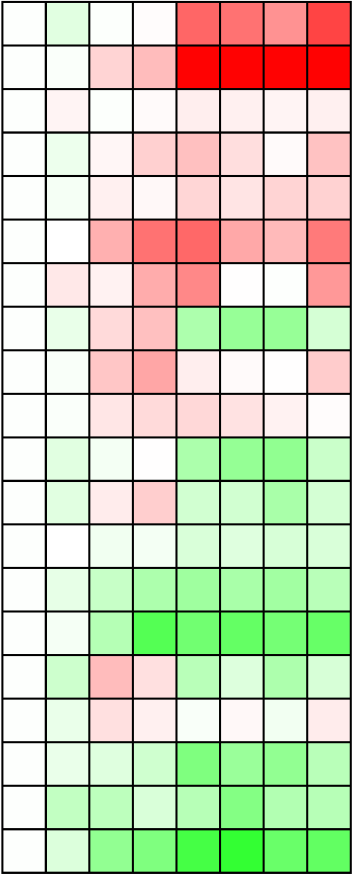


Genes

*PDI1*

*EMC1*

*EMC2*

*EMC3*

*EMC4*

*EMC5*

*EMC6*

*ERO1*

*EUG1*

*IRE1*

*KAR2*

*HAC1*

*YAP1*

*SOD1*

*SOD2*

*CTT1*

*CTA1*

*TSA1*

*TSA2*

*AHP1*

Protein folding

Oxidative stress response

**b**

**a**


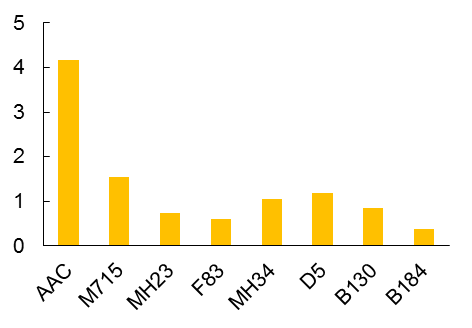


ROS / α-amylase yield


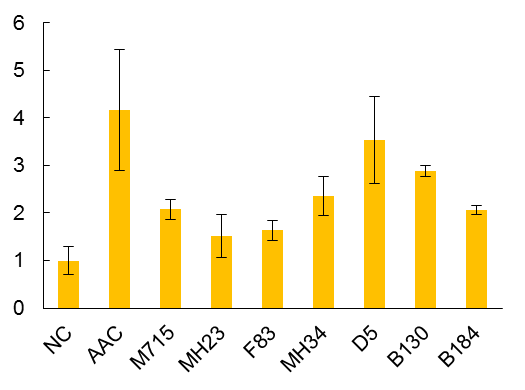


Relative fluorescence unit

(RFU)

Supplementary Figure 12. Reduced oxidative stress in mutant strains. (a) Expression levels of genes related to protein folding in ER and oxidative stress response. (b) Quantification of ROS in strains by DHR123 staining, data shown are mean values ± standard deviations of duplicates. NC: α-amylase non-production strain. (c) Intracellular ROS per α-amylase yield. α-amylase yield of AAC in Fig. 1c was set as 1 and the relative α-amylase yield of other strains in Fig. 1c was calculated correspondingly. ROS per α-amylase yield is calculated by the RFU from Fig. S8b divided by the relative amylase yield. (d) Strains stained by DHR123 were observed using fluorescence microscopy.

NC

AAC

M715

MH23

F83

MH34

D5

B130

B184


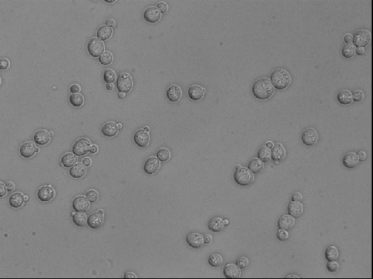

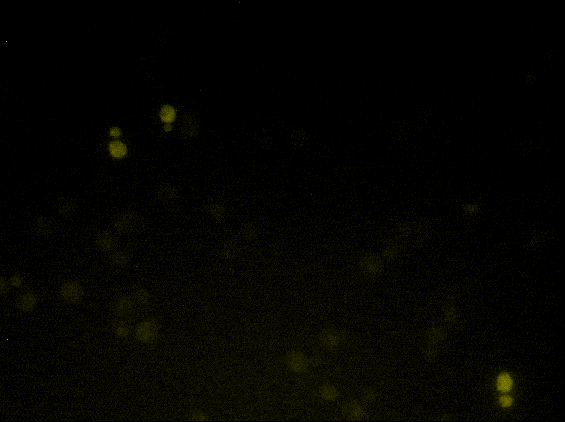

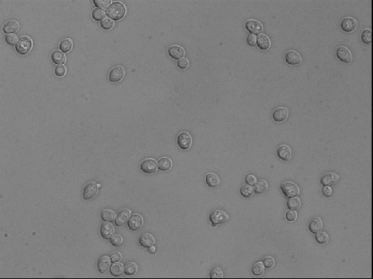

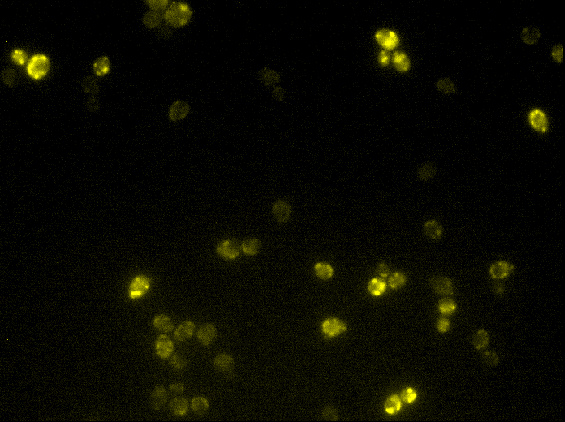

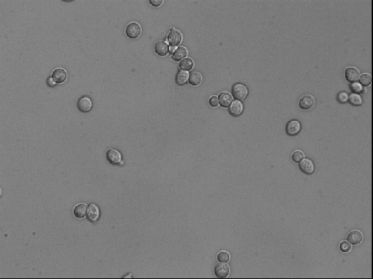

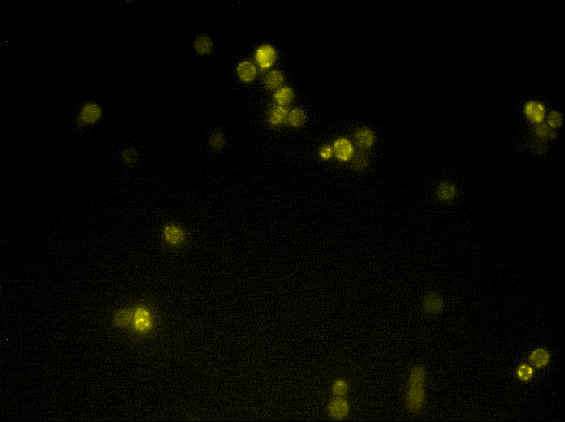

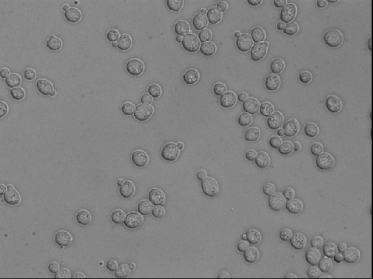

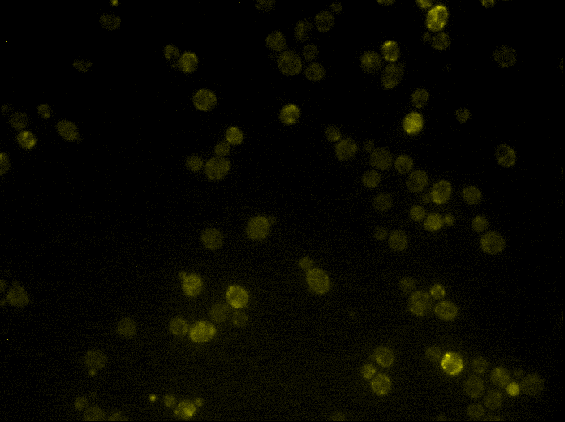

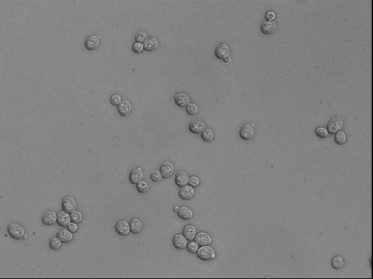

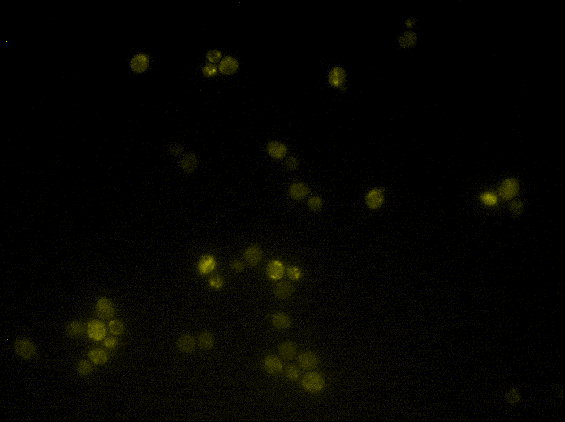

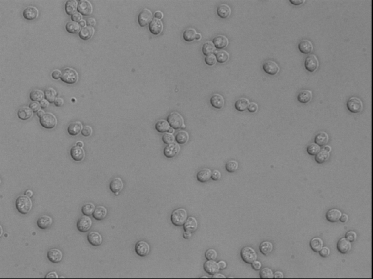

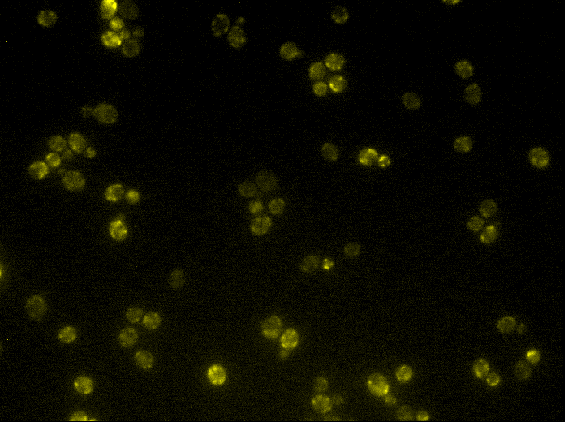

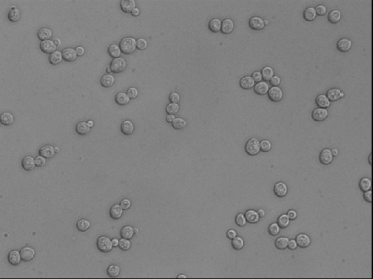

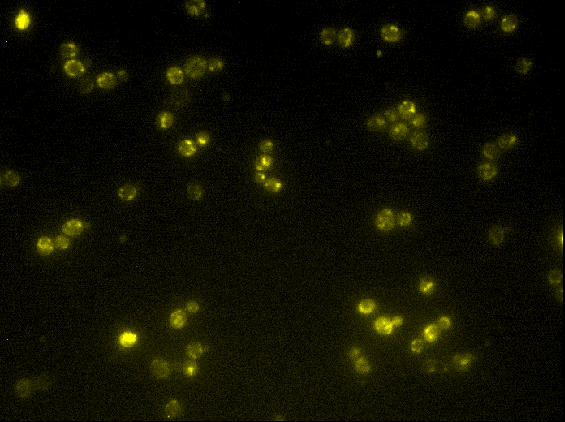

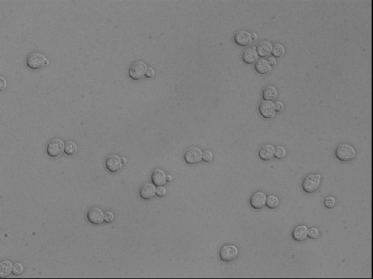

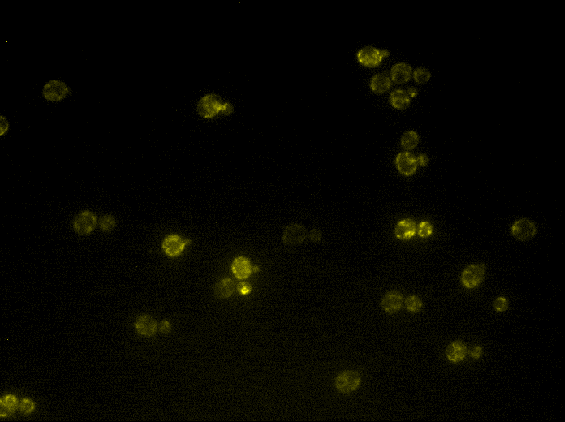

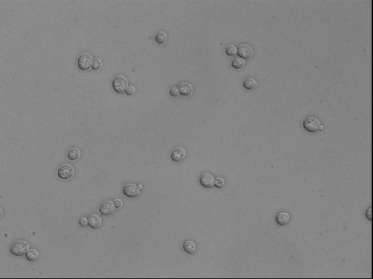

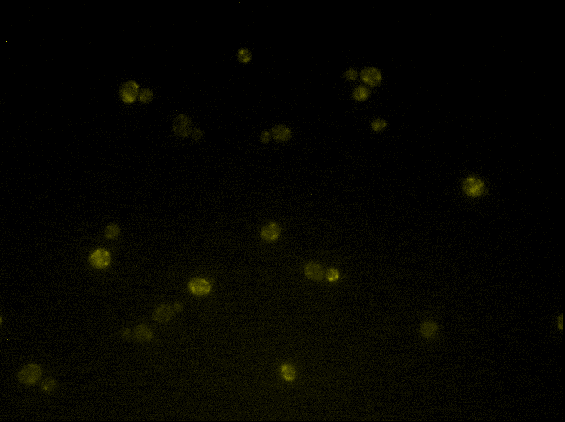


**d**

0

50

μm

Supplementary Fig. 12d. Strains stained by DHR123 were observed using fluorescence microscopy.


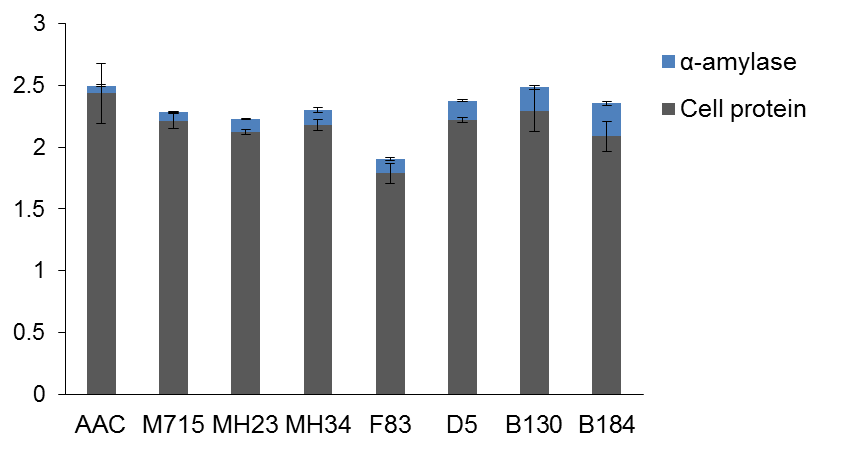


Protein content (g L^-1^)

Supplementary Figure 13. Total protein (yeast cell protein and amylase) didn’t increase in mutant strains compared with the reference strain AAC. The coefficient of 450 mg cell protein per g-DCW was used for calculation of total cell protein from DCW. Data shown are mean values ± standard deviations of triplicates or quadruplicates.

Supplementary Figure 14. A schematic diagram of expression plasmid construction. genes: *MBP1, MSS11, SWI4 and SUT1.*


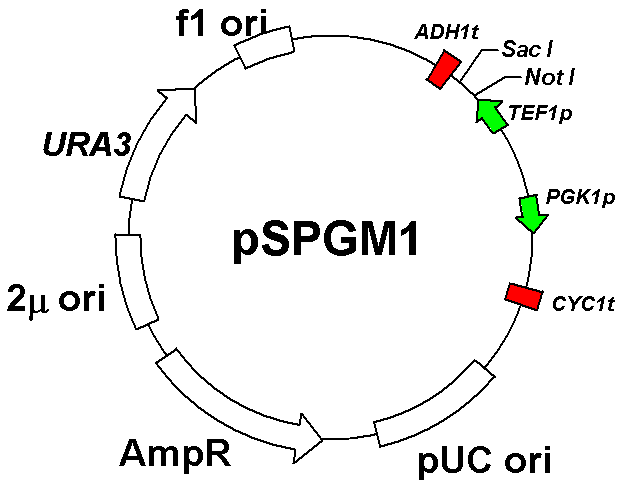

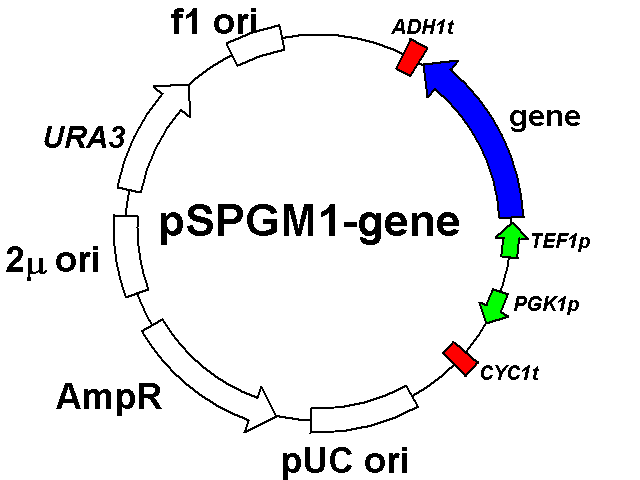


*Not I*

*Sac I*

gene

Restriction enzyme digestion and ligation

Supplementary Table 1. Yield of biomass (X), ethanol (E), glycerol (G), acetate (A) and CO_2_ from glucose (S) (g g^-1^). q_CO2_: Specific CO_2_ production rate (m mol g-DCW^-1^ h^-1^). q_O2_: Specific O_2_ consumption rate (m mol g-DCW^-1^ h^-1^). Data shown are mean values ± standard deviations of triplicates or quadruplicates.

| Strain | Y_SX_ | Y_SE_ | Y_SG_ | Y_SA_ | Y_CO2_ | q_CO2_ | q_O2_ |
| --- | --- | --- | --- | --- | --- | --- | --- |
| AAC | 0.150 ± 0.0038 | 0.231 ± 0.0041 | 0.074 ± 0.0030 | 0.033 ± 0.0007 | 0.444 ± 0.0111 | 6.32 ± 0.188 | 11.51 ± 1.477 |
| M715 | 0.176 ± 0.0121 | 0.264 ± 0.0064 | 0.074 ± 0.0028 | 0.035 ± 0.0009 | 0.482 ± 0.0372 | 6.64 ± 0.276 | 11.16 ± 1.122 |
| MH23 | 0.157 ± 0.0013 | 0.300 ± 0.0185 | 0.068 ± 0.0008 | 0.040 ± 0.0008 | 0.552 ± 0.0014 | 9.80 ± 0.274 | 16.03 ± 0.552 |
| F83 | 0.129 ± 0.0034 | 0.278 ± 0.0072 | 0.073 ± 0.0035 | 0.038 ± 0.0017 | 0.503 ± 0.0481 | 10.16 ± 0.979 | 11.61 ± 1.023 |
| MH34 | 0.155 ± 0.0058 | 0.339 ± 0.0333 | 0.066 ± 0.0076 | 0.027 ± 0.0032 | 0.468 ± 0.0650 | 7.39 ± 0.875 | 7.23 ± 0.363 |
| D5 | 0.115 ± 0.0031 | 0.290 ± 0.0067 | 0.063 ± 0.0021 | 0.021 ± 0.0006 | 0.379 ± 0.0134 | 8.24 ± 0.044 | 12.82 ± 1.231 |
| B130 | 0.119 ± 0.0090 | 0.318 ± 0.0066 | 0.055 ± 0.0037 | 0.020 ± 0.0013 | 0.387 ± 0.0450 | 8.65 ± 0.418 | 8.35 ± 1.025 |
| B184 | 0.126 ± 0.0087 | 0.274 ± 0.0123 | 0.070 ± 0.0053 | 0.026 ± 0.0011 | 0.396 ± 0.0389 | 7.24 ± 0.461 | 9.78 ± 0.471 |

Supplementary Table 2. Amino acid monomer composition of α-amylase and *S. cerevisiae.*

|  | Amino acid monomer composition | | |
| --- | --- | --- | --- |
| Amino acid | mmol per g_α-amylase | mmol per g_cell protein* | Fold^#^ |
| Alanine | 0.607 | 1.020 | 0.60 |
| Arginine | 0.180 | 0.358 | 0.50 |
| Asparagine | 0.426 | 0.227 | 1.88 |
| Aspartic acid | 0.689 | 0.660 | 1.04 |
| Cysteine | 0.148 | 0.016 | 9.25 |
| Glutamine | 0.279 | 0.233 | 1.20 |
| Glutamic acid | 0.197 | 0.671 | 0.29 |
| Glycine | 0.689 | 0.644 | 1.07 |
| Histidine | 0.115 | 0.147 | 0.78 |
| Isoleucine | 0.459 | 0.429 | 1.07 |
| Leucine | 0.558 | 0.658 | 0.85 |
| Lysine | 0.328 | 0.636 | 0.52 |
| Methionine | 0.148 | 0.113 | 1.31 |
| Phenylalanine | 0.213 | 0.298 | 0.71 |
| Proline | 0.344 | 0.367 | 0.94 |
| Serine | 0.590 | 0.411 | 1.44 |
| Threonine | 0.672 | 0.424 | 1.58 |
| Tryptophan | 0.164 | 0.062 | 2.65 |
| Tyrosine | 0.558 | 0.227 | 2.46 |
| Valine | 0.476 | 0.589 | 0.81 |

*Amino acid contents in the biomass of *S. cerevisiae* were based on the data from the reference by Oura^1^. The coefficient of 0.45 g cell protein/gDCW from the reference by Forster et al.^2^ was used to calculate amino acid composition in cell protein of *S. cerevisiae*.

^#^ Amino acid requirement change (fold) in production of α-amylase vs. production of yeast cell protein.

Supplementary Table 3. Total protein concentration and required amounts of cysteine for synthesis of total protein. Data shown are mean values ± standard deviations of triplicates or quadruplicates.

| Strain | Cell protein* and α-amylase (g L^-1^) | Cysteine content in cell protein and amylase (mg L^-1^) | Cysteine requirement for synthesis of total protein (mg gDCW^-1^) |
| --- | --- | --- | --- |
| AAC | 2.49 ± 0.25 | 5.7 ± 0.5 | 1.05 ± 0.10 |
| M715 | 2.28 ± 0.06 | 5.5 ± 0.2 | 1.12 ± 0.04 |
| MH23 | 2.22 ± 0.02 | 5.9 ± 0.1 | 1.26 ± 0.02 |
| MH34 | 2.30 ± 0.06 | 6.3 ± 0.4 | 1.30 ± 0.08 |
| F83 | 1.90 ± 0.09 | 5.4 ± 0.3 | 1.37 ± 0.08 |
| D5 | 2.38 ± 0.03 | 7.0 ± 0.2 | 1.42 ± 0.04 |
| B130 | 2.48 ± 0.19 | 7.7 ± 0.6 | 1.50 ± 0.12 |
| B184 | 2.35 ± 0.14 | 8.7 ± 0.5 | 1.87 ± 0.12 |

* Cell protein was calculated from biomass by using the coefficient of 0.45 g cell protein per gDCW.

Supplementary Table 4. Plasmids and strains used in this study.

| Plasmids and strains | Relevant genotype | Reference |
| --- | --- | --- |
| Plasmids |  |  |
| CPOTud | 2 μm, AmpR, *TPI1*p, *TPI1*t, *POT1* gene from *S. pombe* as a selection marker. | ^3^ |
| pAlphaAmyCPOT | CPOTud-(*TPI1p-alpha factor leader-amylase gene-TPI1t*) | ^3^ |
| pCP-AHSA | CPOTud-(*TPI1p-alpha factor leader-human serum albumin gene-TPI1t*) | This study |
| pCP-AXYN2 | CPOTud-(*TPI1p-alpha factor leader-T. reesei endo-1,4-beta-xylanase II gene-TPI1t*) | This study |
| pSPGM1 | 2 μm, AmpR, *URA3*, *TEF1*p, *ADH1*t, *PGK1*p*, CYC1*t | ^4^ |
| pSPGM1-MBP1 | pSPGM1-( *TEF1*p-MBP1-*ADH1*t) | This study |
| pSPGM1-MSS11 | pSPGM1-( *TEF1*p-MSS11-*ADH1*t) | This study |
| pSPGM1-SWI4 | pSPGM1-( *TEF1*p-SWI4-*ADH1*t) | This study |
| pSPGM1-SUT1 | pSPGM1-( *TEF1*p-SUT1-*ADH1*t) | This study |
| pUG-amdSYM | AmpR, *TEF2*p-amdS-*TEF2*t | ^5^ |
| Strains |  |  |
| CEN.PK 530-1C | *MATa URA3 HIS3 LEU2 TRP1 SUC2 MAL2-8^c^ tpi1(41-707)::loxP-KanMX4-loxP* | ^3^ |
| CEN.PK 530-1CK | *MATa URA3 HIS3 LEU2 TRP1 SUC2 MAL2-8^c^ tpi1(41-707)::loxP* | ^6^ |
| CEN.PK 530-1D | *MATa HIS3 LEU2 TRP1 SUC2 MAL2-8^c^ ura3-52 tpi1(41-707)::loxP-KanMX4-loxP* | ^7^ |
| NC | CEN.PK 530-1C/CPOTud | ^3^ |
| AAC | CEN.PK 530-1C/pAlphaAmyCPOT | ^3^ |
| M715 | derived from AAC by UV mutagenesis | ^8^ |
| MH23 | derived from M715 by UV mutagenesis | ^9^ |
| MH34 | derived from M715 by UV mutagenesis | ^9^ |
| F83 | derived from MH23 by UV mutagenesis | ^9^ |
| D5 | derived from MH34 by UV mutagenesis | ^9^ |
| B130 | derived from MH34 by UV mutagenesis | ^9^ |
| B184 | derived from MH34 by UV mutagenesis | ^9^ |
| B184M | Strain B184 without plasmid pAlphaAmyCPOT | ^9^ |
| AACD | CEN.PK 530-1D/pAlphaAmyCPOT+ pSPGM1 | This study |
| MBP1 | CEN.PK 530-1D/pAlphaAmyCPOT+ pSPGM1-MBP1 | This study |
| MSS11 | CEN.PK 530-1D/pAlphaAmyCPOT+ pSPGM1-MSS11 | This study |
| SWI4 | CEN.PK 530-1D/pAlphaAmyCPOT+ pSPGM1-SWI4 | This study |
| SUT1 | CEN.PK 530-1D/pAlphaAmyCPOT+ pSPGM1-SUT1 | This study |
| 1CH | CEN.PK 530-1C/pCP-AHSA | This study |
| 1CX | CEN.PK 530-1C/pCP-AXYN2 | This study |
| B184H | B184M/pCP-AHSA | This study |
| B184X | B184M/pCP-AXYN2 | This study |
| AAC *aro10Δ* | AAC *aro10Δ::amdSYM* | This study |
| AAC *bas1Δ* | AAC *bas1Δ::amdSYM* | This study |
| AAC *hap2Δ* | AAC *hap2Δ::amdSYM* | This study |
| AAC *hap4Δ* | AAC *hap4Δ::amdSYM* | This study |
| AAC *ilv2Δ* | AAC *ilv2Δ::amdSYM* | This study |
| AAC *kgd1Δ* | AAC *kgd1Δ::amdSYM* | This study |
| AAC *thi2Δ* | AAC *thi2Δ::amdSYM* | This study |
| AAC *thi3Δ* | AAC *thi3Δ::amdSYM* | This study |
| AAC *thi4Δ* | AAC *thi4Δ::amdSYM* | This study |
| AAC *tup1Δ* | AAC *tup1Δ::amdSYM* | This study |
| AACK | CEN.PK 530-1CK/pAlphaAmyCPOT | ^6^ |
| AACK *pgm2Δ* | AACK *pgm2Δ::amdSYM* | This study |
| AACK *pxa1Δ* | AACK *pxa1Δ::amdSYM* | This study |
| AACK *FBA1p-PDI1* | AACK *amdSYM-FBA1p-PDI1* | This study |
| AACK *emc1Δ* | AACK *emc1Δ::amdSYM* | This study |
| AACK *uso1Δ* | AACK *uso1Δ::amdSYM* | This study |
| AACK *vps10Δ* | AACK *vps10Δ::amdSYM* | This study |
| B184 *thi2Δ* | B184 *thi2Δ::amdSYM* | This study |
| B184 *thi3Δ* | B184 *thi3Δ::amdSYM* | This study |
| B184 *thi4Δ* | B184 *thi4Δ::amdSYM* | This study |

Supplementary Table 5. Primers used in this study.

| Name | Sequence (5’→3’)^#^ |
| --- | --- |
| Plasmid construction | |
| MBP1EP1 | ACTGCGGCCGCAACAAAATGTCTAACCAAATATACTCAGCGAGAT |
| MBP1EP2 | CGTGAGCTCTTATGCATGACTATTCGCGTTTGAGA |
| MSS11EP1 | ACTGCGGCCGCAACAAAATGGATAACACGACCAATATTAATACAAATGAG |
| MSS11EP2 | CGTGAGCTCTTAGCTATCCATTAGATCAGGAGAAAAGTC |
| SWI4EP1 | ACTGCGGCCGCAACAAAATGCCATTTGATGTTTTGATATCAAATCAAAAAG |
| SWI4EP2 | CGTGAGCTCTTATGCGTTTGCCCTCAAATCC |
| SUT1EP1 | ACTGCGGCCGCAACAAAATGTCCACAAGCATTACAGTAAGAA |
| SUT1EP2 | CGTGAGCTCCTAAAAATCAATGCTTTTATAGTCATCATAGGT |
| Deletion cassette and promoter replacement cassette construction | |
| ARO10F | *ATGGCACCTGTTACAATTGAAAAGTTCGTAAATCAAGAAGAACGACACCTTGTTTCCAACCGATCAGCAACAATTCCGTTTGGTG*gacatggaggcccagaatac |
| ARO10R | *CAGAAAACGAACAATTGGTAGCAGTGTTTTATAATTGCGCCCACAAGTTTCTA***GCTTAAGGGAGTTTCTTTGTTATCTTGTAAATAAACTTTACAGAA**cagtatagcgaccagcattc |
| BAS1F | *ATGTCGAATATAAGTACCAAAGATATACGAAAAAGTAAGCCAAAAAGAGGATCCGGCTTCGATTTACTTGAAGTGACTGAATCAC*gacatggaggcccagaatac |
| BAS1R | *CTAATATGTTAAACAATTGAAAGATTTGTGTTTTTTTTTCGGCCTTGCCTTCTCA***TCTCGATAAAATGTATTCTGCGATAAAAAAAAAAGAGCAAAAACG**cagtatagcgaccagcattc |
| GCR1F | *ATGAATTTTCTGACTCAGGCTATGTCAGAAACTTTTCAAGGGACAAATAACAGGATAAAACGTAATGTCAGGACACAAAGTGTGC*gacatggaggcccagaatac |
| GCR1R | *GCTTCGTTATTTTGTTGAAGGAACTATTGTCGCGGACAACCTCAATAAACTTA***CCTCTTGTACGCAGACAGACGCAGACAACACTCAATTGATAGCTA**cagtatagcgaccagcattc |
| HAP2F | *ATGTCAGCAGACGAAACGGATGCGAAATTTCATCCATTAGAAACAGATCTGCAATCTGATACAGCGGCTGCAACATCAACGGCAG*gacatggaggcccagaatac |
| HAP2R | *GCACTTTTAGTTCTTTTTAGGAATGATATTAACATTGGAATATTACAAAATTA***GACGTTCTTGTTCCTCTTCCAAAAAGAAATGCTTTTCCAAATACA**cagtatagcgaccagcattc |
| HAP4F | *ATGACCGCAAAGACTTTTCTACTACAGGCCTCCGCTAGTCGCCCTCGTAGTAACCATTTTAAAAATGAGCATAATAATATTCCAT*gacatggaggcccagaatac |
| HAP4R | *TTTGTTTTCGTGATTTTTAGTTGTTTTCGTTTTATTGCAACATGCCTATTTCA***TTAAAATGCTCTTTGATGTACTAGGAGACCAATAAATATGTGTGG**cagtatagcgaccagcattc |
| ILV2F | *ATGATCAGACAATCTACGCTAAAAAACTTCGCTATTAAGCGTTGCTTTCAACATATAGCATACCGCAACACACCTGCCATGAGAT*gacatggaggcccagaatac |
| ILV2R | *AAGTCTGCATTTTTTACTGAAAATGCTTTTGAAATAAATGTTTTTGAAATTCA***TTGAAAATTGATTCTGTTGTATTTATCTCCTCTTAGCTCAAAGGG**cagtatagcgaccagcattc |
| KGD1F | *ATGCTAAGGTTCGTGTCTTCGCAAACCTGCCGGTATAGTTCAAGAGGACTATTAAAAACATCTTTACTTAAAAATGCATCTACTG*gacatggaggcccagaatac |
| KGD1R | *TACGTGTTTATATGGCTGTATTTTCATGTTTTTCATATTTGAATTCATCTTTA***AACGGTAAAATTTAGTATTTTCTAAAAACTGCTTTCTTTCTCTTT**cagtatagcgaccagcattc |
| THI2F | *ATGATCAATAGTAAGAGGCAGCAGAGAAGCAAGAAAGTAGCGTCATCCTCCAAAGTGCCCCCCACCAAGGGGAGGACATTT*gacatggaggcccagaatac |
| THI2R | *GCTTTTGGCTTTTTTTTTCTTGAAATGAGTGAAGGGAAGGCTCAATAAGCCTA***TTGGTTCTAGTGCGGATATATATATAGGCTATATATATACGTGGT**cagtatagcgaccagcattc |
| THI3F | *ATGAATTCTAGCTATACACAGAGATATGCACTGCCGAAGTGTATAGCAATATCAGATTATCTTTTCCATCGGCTCAACCAGCTG*gacatggaggcccagaatac |
| THI3R | *ATTCGAGCGGTAATCATGAGGGTCCCTGGTAGTAGGGCGGAGAGATCAGATCA***CTTCAGAATGACGACGGTGCGTTTTAGTAGTTATGTTCTTTCAAA**cagtatagcgaccagcattc |
| THI4F | *ATGTCTGCTACCTCTACTGCTACTTCCACAAGTGCCTCTCAATTGCACTTAAACTCTACTCCAGTTACTCACTGCTTATCTGACA*gacatggaggcccagaatac |
| THI4R | *ATAGCGTGTATAAAAAATATATAAATCTTTAAAAGCATACCTCATTAAAAGCCTA***TTTGATAGTTAGTTGATTTTTTTGGTTGTGATTTATTATTTCTAG**cagtatagcgaccagcattc |
| TUP1F | *ATGACTGCCAGCGTTTCGAATACGCAGAATAAGCTGAATGAGCTTCTCGATGCCATCAGACAGGAGTTTCTCCAAGTCTCACAAG*gacatggaggcccagaatac |
| TUP1R | *GAATAGTTTAGTTAGTTACATTTGTAAAGTGTTCCTTTTGTGTTCTGTTCTTA***ATTGGTTTGGATGGAAAGCTGATTTCTTTCTTCCCCTGCTTATCA**cagtatagcgaccagcattc |
| PGM2F | ATGTCATTTCAAATTGAAACGGTTCCCACCAAACCATATGAAGACCAAAAGCCTGGTACCTCTGGTTTGCGACATG GAGGCCCAGA ATAC |
| PGM2R | TTCATTAAAAAAGGTGAAAATCATTAAGCCATTAGTAAATCATTCGTTTTATCAGTTATGTTAACTTTTGTTACTTTTTTTACTGAGAAAGATTGGCAGTATAGCG ACCAGCATTC |
| PXA1F | ATGTCAACAACATTAGCAGCACCAGCGAAACTAAAGAGTTTGCTCCTGAATCTACATACTCACTGTATTGGGCTAGACATG GAGGCCCAGA ATAC |
| PXA1R | ATCTGTACTAGCTCCTTATCTCTAAACCCCCTATCAAAGAATTCATCTCATTATTCTGTGTTATGCGTTCTAAGACTTCCAGATTGTCTGAAACGGGCAGTATAGCG ACCAGCATTC |
| EMC1F | ATGAAGATAACGTGTACAGACTTGGTGTACGTCTTCATTTTACTCTTCCTAAACACGAGTTGTGTCCAAGCCGTTGACATG GAGGCCCAGA ATAC |
| EMC1R | GGAGCCTTGAAATGTGTATCTGATATATATAATGTGTATGTAAATATCTATTATGCTATGGGGGAAGGGGAGGATGAAAGTGTTGATATGAATGTAGCAGTATAGCG ACCAGCATTC |
| USO1F | ATGGACATCATTCAAGGACTGATACAGCAACCAAAAATTCAATCTGTGGATGAAACCATTCCGACGTTGTGCGATGACATG GAGGCCCAGA ATAC |
| USO1R | TTGAGATAATTTCCTCCTTCGTTTGTTCTAAGTCAGAGATGGTTTTTTTATCATCTTGATAGGAGGGTTGTTATAGATATTAATCACTCGAAGTCGTCAGTATAGCG ACCAGCATTC |
| VPS10F3 | ATGATATTACTTCATTTTGTCTATTCTCTTTGGGCCTTACTTCTCATTCCTTTAACTAATGCCGAAGAATTCACCGACATG GAGGCCCAGA ATAC |
| VPS10R4 | TTTATGAAAAGTATATGGAATTATCTACTCTATGTAAAGTAATCTCTCTATTAAACGTGTGATGACTACTGGACACTTCAGGGCTTTTCCAGATATACAGTATAGCG ACCAGCATTC |
| PDIFPR1 | GCATTTTGTTGTGCTGTTACAACCACAACAAAACGAAAAACCCGTATGGATCCAACTGGCACCGCTGGCTTGAACAACAATACCAGCCTTCCAACTTCGACATG GAGGCCCAGA ATAC |
| PDIFPR2 | CAGTATAGCG ACCAGCATTC |
| PDIFPR3 | AAGTTAAGTGCGCAGAAAGTAATATCATGCGTCAATCGTATGTGAATGCTGGTCGCTATACTGTCCAACTGGCACCGCTGGCTT |
| PDIFPR4 | ACAGCCTCTTGTTGGGCGAAAACAGAGGAGGCGAGCAGCAGGGAGGACCATGACAGGACGGCACCAGCAGAAAACTTCATTTTGAATATGTATTACTTGGTTATGGTTATATATGAC |
| Gene deletion and promoter replacement verification | |
| ARO10P1 | CGGTATGTAATAGGTTAGTGGCAT |
| ARO10P2 | TATAGTTCTTCAAGTGGTGAGCCGA |
| BAS1P1 | AGGGTCCAGTCACAGAATAAAGC |
| BAS1P2 | TAAGAAGAGAAATCTTGGAAGAATACCCT |
| GCR1P1 | ACCTACTTGATAAAGCCACATACCTC |
| GCR1P2 | TCCCACATAGGCATACATCCG |
| HAP2P1 | CCAAGCCAACCAGTCATGTAAATATC |
| HAP2P2 | CAGCGGAGTACATAGGTCTGTTAG |
| HAP4P1 | TGCTATCTACAGGTCCACTTTACACT |
| HAP4P2 | GGCCTTCCAGACCCATATTTGTT |
| ILV2P1 | TCTAATCCTTTCTCCACCATCCCT |
| ILV2P2 | ATCAAATACTACCAACTCACTTGAATCGA |
| KGD1P1 | CTGCGGGAGTCAAGACCTAC |
| KGD1P2 | GTGAATACACCAGAGGAGGAAGTAGG |
| THI2P1 | GCACCTTTCTCGGTCGGAATT |
| THI2P2 | ACATAGATATCCGATAAATGTCAGCAAAG |
| THI3P1 | TTTCCCTTTCTTCATACTTTACGGCATAG |
| THI3P2 | GCTAGGCTACTGTTCAGCGAAT |
| THI4P1 | TTTACGAGCACTAATAGAAATGGATCATG |
| THI4P2 | TCTCATCACTATCGGATCTTTCCACTTAG |
| TUP1P1 | CAATTGATAAACATCAGCGAAGCAAG |
| TUP1P2 | GGGAAGGGATGAATGGTGAGGAAAG |
| PGM2P1 | CTGCCGCCCGAACAATGTGG |
| PGM2P2 | GCACTTATGAAGAAGATACTGCGATTA |
| PXA1P1 | CCGCTTGAACCTGTGGAATGT |
| PXA1P2 | TGATACGTGGATTCATCAAGTGAGA |
| EMC1P1 | ATTAGGTCTTTGCGGATTGCT |
| EMC1P2 | CCAGATGTGGTGGATTGAATGAAG |
| USO1P1 | ATTACCAGAAATAAAGCCTAAAGCG |
| USO1P2 | GGCTTCCAGCTCTTCCCTAG |
| VPS10P3 | GAGGCGAGTAGGTGGTACTAT |
| VPS10P4 | AACAACCACTTTCCCGTAACATA |
| PDIPR5 | TGCACGTGATAATATGTTACCCTGTC |
| PDIPR6 | GGAGGAGGATGAGATAAGTAGTTTCC |

^#^ underlined sequence indicates restriction site; italicized sequence indicates homology arm to targeted gene; bolded sequence indicates direct repeat of the region upstream the gene to excise the amdSYM marker upon counter selection; lowercase sequence is used for amplification of amdSYM cassette from the plasmid pUG-amdSYM.

**Supplementary References**

1. Oura, E. The effect of aeration on the growth energetics and biochemical composition of baker’s yeast. *PhD thesis, University of Helsinki, Finland* (1972).

2. Forster, J., Famili, I., Fu, P., Palsson, B.O. & Nielsen, J. Genome-scale reconstruction of the *Saccharomyces cerevisiae* metabolic network. *Genome Res.* **13**, 244-253 (2003).

3. Liu, Z., Tyo, K.E.J., Martínez, J.L., Petranovic, D. & Nielsen, J. Different expression systems for production of recombinant proteins in *Saccharomyces cerevisiae*. *Biotechnol. Bioeng.* **109**, 1259-1268 (2012).

4. Chen, Y., Partow, S., Scalcinati, G., Siewers, V. & Nielsen, J. Enhancing the copy number of episomal plasmids in *Saccharomyces cerevisiae* for improved protein production. *FEMS Yeast Res.* **12**, 598-607 (2012).

5. Solis-Escalante, D. et al. amdSYM, a new dominant recyclable marker cassette for *Saccharomyces cerevisiae*. *FEMS Yeast Res.* **13**, 126-139 (2013).

6. Bao, J., Huang, M., Petranovic, D. & Nielsen, J. Moderate expression of SEC16 increases protein secretion by *Saccharomyces cerevisiae*. *Appl. Environ. Microbiol.* (2017).

7. Hou, J., Tyo, K., Liu, Z., Petranovic, D. & Nielsen, J. Engineering of vesicle trafficking improves heterologous protein secretion in *Saccharomyces cerevisiae*. *Metab. Eng.* **14**, 120-127 (2012).

8. Liu, Z. et al. Improved Production of a Heterologous Amylase in *Saccharomyces cerevisiae* by Inverse Metabolic Engineering. *Appl. Environ. Microbiol.* **80**, 5542-5550 (2014).

9. Huang, M. et al. Microfluidic screening and whole-genome sequencing identifies mutations associated with improved protein secretion by yeast. *Proc. Natl Acad. Sci. USA* **112**, E4689–E4696 (2015).
