# Supplementary material for: Efficient protein production by yeast requires global tuning of metabolism
Source: Nat Commun. 2017 Oct 25;8:1131. doi: 10.1038/s41467-017-00999-2 (PMC5656615; doi:10.1038/s41467-017-00999-2)
Supplement: Supplementary file 3 — Description of Additional Supplementary Files [file 41467_2017_999_MOESM3_ESM.pdf]

Description of Additional Supplementary Files

File Name: Supplementary Data 1
Description: GO Slim mapper analysis of mutant genes and differentially expressed genes (p-adj<0.05) in mutant strains.
